# Aspartyl tRNA-synthetase (AspRS) gene family enhances drought tolerance in poplar through BABA-*PtrIBIs*-*PtrVOZ* signaling module

**DOI:** 10.1186/s12864-023-09556-2

**Published:** 2023-08-21

**Authors:** Cong-Hua Feng, Meng-Xue Niu, Shilei Zhao, Shangjing Guo, Weilun Yin, Xinli Xia, Yanyan Su

**Affiliations:** 1 College of Agronomy, Liaocheng University, Liaocheng, 252000 China; 2 National Engineering Laboratory for Tree Breeding, College of Biological Sciences and Technology, Beijing Forestry University, Beijing, 100083 China

**Keywords:** Aspartyl tRNA-synthetase, Poplar, BABA, Drought stress

## Abstract

**Background:**

Drought stress is a prevalent abiotic stress that significantly hinders the growth and development of plants. According to studies, β-aminobutyric acid (BABA) can influence the ABA pathway through the *AtIBI1* receptor gene to enhance cold resistance in *Arabidopsis*. However, the Aspartate tRNA-synthetase (AspRS) gene family, which acts as the receptor for BABA, has not yet been investigated in poplar. Particularly, it is uncertain how the AspRS gene family (*PtrIBIs*)r can resist drought stress after administering various concentrations of BABA to poplar.

**Results:**

In this study, we have identified 12 AspRS family genes and noted that poplar acquired four *PtrIBI* pairs through whole genome duplication (WGD). We conducted cis-action element analysis and found a significant number of stress-related action elements on different *PtrIBI* genes promoters. The expression of most *PtrIBI* genes was up-regulated under beetle and mechanical damage stresses, indicating their potential role in responding to leaf damage stress. Our results suggest that a 50 mM BABA treatment can alleviate the damage caused by drought stress in plants. Additionally, via transcriptome sequencing, we observed that the partial up-regulation of BABA receptor genes, *PtrIBI2/4/6/8/11*, in poplars after drought treatment. We hypothesize that poplar responds to drought stress through the BABA-*PtrIBIs-PtrVOZ* coordinated ABA signaling pathway. Our research provides molecular evidence for understanding how plants respond to drought stress through external application of BABA.

**Conclusions:**

In summary, our study conducted genome-wide analysis of the AspRS family of *P. trichocarpa* and identified 12 *PtrIBI* genes. We utilized genomics and bioinformatics to determine various characteristics of *PtrIBIs* such as chromosomal localization, evolutionary tree, gene structure, gene doubling, promoter cis-elements, and expression profiles. Our study found that certain *PtrIBI* genes are regulated by drought, beetle, and mechanical damage implying their crucial role in enhancing poplar stress tolerance. Additionally, we observed that external application of low concentrations of BABA increased plant drought resistance under drought stress. Through the BABA-*PtrIBIs-PtrVOZ* signaling module, poplar plants were able to transduce ABA signaling and regulate their response to drought stress. These results suggest that the *PtrIBI* genes in poplar have the potential to improve drought tolerance in plants through the topical application of low concentrations of BABA.

**Supplementary Information:**

The online version contains supplementary material available at 10.1186/s12864-023-09556-2.

## Background

The impact of drought stress on plant production is increasing due to the worsening greenhouse effect caused by global climate change [1, 2]. Drought stress is one of the most common abiotic stressors that can cause substantial damage to plant growth. Plants subjected to drought stress experience reduced gas exchange, chlorophyll content, photosynthesis, and water use efficiency, and are more likely to produce reactive oxygen species (ROS) [3–6]. Moreover, drought stress can alter the levels of plant hormones, such as abscisic acid (ABA), jasmonic acid, salicylic acid, and cytokinin [7–9]. ABA plays a critical role as an anti-stress hormone, and it promotes stomatal closure to regulate plant water balance and osmotic homeostasis in response to drought stress [7, 10]. Therefore, ABA-mediated regulation of osmotic pressure is a vital aspect of the plant’s response to drought stress [1]. To activate the ABA signaling pathway and initiate the stress response process, the ABA receptor *PYR1* and Pyr1-like proteins (*PYL*) bind ABA, and interact with *PP2C* to activate the *SnRK/PP2C* complex [11–13]. The activated *SnRK2* then phosphorylates downstream ion channels and transcription factors (TFs), leading to upregulation of various genes, including NACs, MYBs, LEAs, and WRKYs by targeting the ABA response elements (ABREs) in the promoter regulatory region. These TFs eventually activate the ABA signaling pathway and the process of stress response [14–17].

The use of specific chemicals can increase the ability of plants to withstand abiotic stresses, with β-aminobutyric acid (BABA) serving as an important initiator that offers protection against a wide range of diseases [18]. The study of immune priming in plants has become increasingly popular with the use of β-aminobutyric acid-IR (BABA-IR) as a model system, allowing researchers to investigate the molecular mechanisms involved [19, 20]. Researchers have found that applying BABA topically can enhance a plant’s resistance to biotic stresses [21]. BABA has been shown to induce enhanced defense responses through the immune system of various plants such as *Capsicum annuum*, *Potato*, *Arabidopsis*, *Hordeumvulgare L*, *Nicotiana tabacum L*, *Peach*, amongst others, by initiating a salicylic acid (SA)-dependent defense mechanism, leading to a stronger resistance to diseases [22–27]. Additionally, BABA application can significantly increase the activity of several defense enzymes against *Aphis glycines* in wheat and soybean [25, 28]. It has also been observed that BABA application can result in the reduction of *Sitobion avenae* performance on wheat seedlings, and it has been suggested that the mechanism behind this effect is the direct toxicity of high BABA contents in the plant’s phloem [29].

BABA not only enhances plant resistance to diseases, but also to abiotic stresses [7]. It plays an important role in enhancing salt stress tolerance in *Brassica napus L* and soybean by upregulating antioxidant defense. It also attenuates damage from cadmium stress in soybean [30, 31]. Additionally, BABA has been found to enhance chilling resistance in tobacco and is closely associated with Ca^2+^ signaling status [32]. BABA triggers the accumulation of ABA, reduces reactive oxygen species (ROS) production, and increases antioxidant defense enzymes, thereby improving drought tolerance in wheat and maize [33–35]. *Arabidopsis thaliana* also shows increased drought, salinity, and heat stress resistance after BABA treatment [7, 19, 36, 37]. In *Arabidopsis*, *AtIBI1* acts as a receptor for BABA and encodes an aspartate tRNA synthetase (AspRS). BABA activates *AtIBI1*, which controls plant immunity and growth. *AtVOZ1* and *AtVOZ2* transcription factors (TF) interact with *AtIBI1* and are induced by ABA transcription. They negatively regulate *Arabidopsis thaliana* response to cold stress [38–40]. Several studies have shown that exogenous BABA seems to confer the ability of plants to resist stress, but the role of BABA and *PtrIBIs* in poplar drought tolerance is unclear.

Here, we identified the BABA receptor Aspartyl tRNA-synthetase (AspRS) gene family in poplar. And we used genomics and bioinformatics to determine the chromosomal localization, evolutionary tree, gene structure, gene doubling, promoter cis-elements, and expression profiles of *PtrIBIs*. Under drought stress, external application of low concentrations of BABA increased drought resistance in poplar. We used bioinformatics to discover that the TF-*PtrIBIs* module plays a crucial role in regulating plant responses to drought stress. Finally, the BABA-*PtrIBI*-*PtrVOZ* signalling module was analysed in conjunction with transcriptomic data, and the results indicate that the regulation of this module plays an important role in the response to drought stress in poplar.

## Methods

### Identification and protein property analysis of AspRS gene family in *P. trichocarpa*

To predict the protein sequences of *PtrIBIs*, the *P. trichocarpa* 4.0 genome was searched using HMMER and the Hidden Markov model of Aspartyl tRNA-synthetase (PF00152) in the Pfam 35.0 database (http://pfam.xfam.org/, accessed on 22 April 2022) was used as a query [41]. The protein BLAST database (https://blast.ncbi.nlm.nih.gov/Blast.cgi, accessed on 23 April 2022) was utilized to confirm the *PtrIBI* genes family. Extraction of the *P. trichocarpa* 4.0 genome, CDS, transcripts, amino acids, and 2000 bp upstream of the ATG promoter region was completed from the Phytozome 13.0 database (https://phytozome-next.jgi.doe.gov/, accessed on 23 April 2022). *PtrIBIs* are named according to their position on the chromosome. Gene locations and chromosome sizes of *PtrIBIs* were obtained from the NCBI database (https://www.ncbi.nlm.nih.gov/, accessed on 25 April 2022) and visualized by TBtools (TBtools_windows-x64_1_098748) Gene Location Visualize (South China Agricultural University, Guangzhou, China) [42]. Protein physicochemical property prediction was performed using the ProtParam website (http://www.expasy.org/tools/protparam.html accessed on 28 April 2022) [43]. *PtrIBIs* gene family subcellular localization prediction was performed via the Plant-mPLoc website (http://www.csbio.sjtu.edu.cn/bioinf/plant-multi/, accessed on 30 April 2022) [44].

### Multiply alignments and phylogenetic analysis

We constructed phylogenetic tree in eight species of *PtrIBIs*, *AtIBIs* and *OsIBIs* by pairwise deletion and 1000 bootstraps replicates using the Neighbor-Joining (NJ) method parameter on MEGA7.0 [45]. To show the evolutionary relationships more clearly, the phylogenetic trees were visualized using the iTOL online program (https://itol.embl.de/, accessed on 30 April 2022) [46]. We used the ClustalW website (https://www.genome.jp/tools-bin/clustalw, accessed on 30 May 2022) to perform the AspRS sequence comparison among different species using the Clustal algorithm to obtain the clustalw aln file [47]. We used the ENDscript/ESPript website (https://espript.ibcp.fr/ESPript/cgi-bin/ESPript.cgi, accessed on 30 May 2022) for column comparison for mapping.

### Analysis of conserved motifs, conserved structural domains and gene structures

We provided the gff3 annotation file with *PtrIBI* genes family ID numbers to TBtools software to reveal the gene structure. Next we submitted the *PtrIBIs* gene family protein sequences to the web version of Multiple Expectation Maximization (https://meme-suite.org/meme/tools/meme, accessed on 30 May 2022) for Motif (MEME) [48]. The number of motifs in the default parameters is changed to 10, and the other parameters remain the same by default. We submitted the protein sequences to the NCBI database to obtain hitdata files. In addition, phylogenetic trees stored in Newick format, motifs stored in Xtensible Markup Language (XML) format, hitdata files describing conserved structural domains, and gene structures stored in gff3 format were provided to TBtools software for displaying phylogenetic trees, conserved motifs and gene structures.

### Gene colinearity analysis and identification of gene duplication events

We performed a one-step MCScanX study of putative replication events using TBtools and default parameters applying poplar genome fast files and gff3 files [49]. In addition, non-synonymous (Ka) and synonymous (Ks) substitution rates of *PtrIBI* gene pairs were determined to assess the selection pressure during the evolution of *PtrIBIs* [50]. We further evaluated the genetic covariance of *P. trichocarpa* with other plants. We used the default parameters of MCScanX to identify putative direct homologs. We compared genomic FAST files and gff3 files of *P. trichocarpa* with other plants and obtained three important files: control file (ctl), gff and collinearity formats. Unnamed chloroplasts and mitochondria were first manually removed from the ctl files and reordered, and finally visualized by TBtools [51].

### Analysis of cis-regulatory elements of ***PtrIBIs***

The cis-acting elements of the promoters of the *PtrIBI* genes family were predicted by the PlantCARE website (http://bioinformatics.psb.ugent.be/webtools/plantcare/html/, accessed on 28 May 2022). We performed a classification analysis in EXCEL based on the literature and visualized the results using EXCEL and TBtools.

### Transcriptome analysis and visualization of the *PtrIBI* genes family

To analyze the gene expression patterns of the *PtrIBIs* family, we downloaded the transcriptome data of the *PtrIBI* genes family from the PopGenIE (https://popgenie.org/, accessed on 24 May 2022) public database [52–54]. In this database, expression data were collected for 15 different plant tissues, as well as for three abiotic stresses and one biotic stress. Heat map of *PtrIBIs* is drawn with TBtools, and choose row scale for homogenization, all other parameters are default.

### Plant materials and treatments

*Populus tomentosa* was used as the experimental material, were cultivated in a woody plant medium (WPM) supplement with 0.05mg/L 1-Naphthylacetic acid (NAA) (pH 5.8) [55]. With a 16h light/8h dark photoperiod, the poplar varieties were propagated in the greenhouse at 23°C and 74% humidity. One month later, transplant the tissue culture seedlings into nutrient soil. Forty seedlings of the same genotype were selected from wild-type *P. tomentosa* with similar growth vigor, divided into four groups (10 plants each in the control, drought stress, 50 mM BABA drought treatment, and 200 mM BABA drought treatment), and incubated for two months. Poplars were subjected to drought treatment, and the control and drought stress treatment groups were externally treated with the same amount of water as the BABA treatment group. Leaves and roots were removed from poplar plants after 15 days of drought treatment and stored in an ultra-low temperature refrigerator at -80°C [56].

### Quantitative real-time (qRT-PCR) analysis

Our *Populus tomentosa* transcriptome data obtained by high-throughput sequencing for drought treatment and drought under different concentrations of BABA treatment (Supplemental Table S9). It was used to extract *PtrIBI* genes expression and visualize the expression heat map using TBtools software.

For further analysis of *PtrIBI* genes expression patterns, the total RNA of collected samples was extracted with an RNA extraction kit (Vazyme Biotech Co. Ltd. Beijing, China). Then, the FastKing RT kit (kit from TIANGEN BIOTECH CO. LTD. Beijing, China) was used to synthesize first-strand cDNA of the total RNA. The *PtrIBI* genes primers for qRT-PCR were designed according to the NCBI Primer-BLAST online tool (https://blast.ncbi.nlm.nih.gov/Blast.cgi, accessed on 30 January 2023) (Supplemental Table S11) [57].

The qRT-PCR was run with the CFX96 Touch™ instrument (Bio-Rad Co. Ltd. Hercules, CA, USA) to detect the chemical SYBR Green. The following qRT-PCR procedure was used: the template melting at 95°C for 15min, followed by amplification for 45 cycles with a denaturation temperature of 95°C for 10s, an annealing temperature of 58°C for 30s, and an extending temperature of 72°C for 30s. Quantitative analysis of *PtrIBI* genes expression was performed according to the 2^−∆∆CT^ method and the *PtrUBQ* was considered as the internal control [58, 59].

### Bioinformatics analysis of potential TFs in the upstream region of *PtrIBIs*

Possible upstream transcription factors of *PtrIBIs* were identified using the PlantRegMap database (http://plantregmap.gao-lab.org/network.php, accessed on 26 January 2023) [60]. Network structure maps of target genes and upstream TFs were visualized using PowerPoint. Early in the study, gene expression profiles of upstream TFs under different treatments were obtained from poplar transcriptome data by high-throughput sequencing.

### Statistical analyses

We analyzed the experimental data with Microsoft Excel 2020 (Microsoft Corporation, Redmond, WA, USA) and SPSS v.25.0 (SPSS Inc., Chicago, IL, USA). One-way ANOVA with the LSD multiple comparisons test (*p < 0.05, **p < 0.01.) was performed for the gene relative expression. Before applying the ANOVA test, the data were tested for normality and homogeneity of variance. Student‘s t test (*p < 0.05, **p < 0.01.) was performed for the leaf RWC, relative electrical conductance(REC) etc.

## Results

### Genome-wide Identification of *PtrIBI* Genes in *P. trichocarpa*

We searched for the conserved Aspartyl tRNA-synthetase (AspRS) (PF00152) structural domain in the genome-wide protein database of *P. trichocarpa*. The motif map of the structural domains (Fig. S1) and the hmm model file were used to finally filter out 19 sequences of *P. trichocarpa*. We obtained a total of 12 *PtrIBI* genes by manually removing redundant sequences and naming the genes according to the order of the corresponding chromosomal positions identified in the NCBI database (Supplementary Table S1). The *PtrIBI* genes are distributed on eight chromosomes in the genome of *P. trichocarpa* (Fig. S2). There were three *PtrIBI* genes on each of chromosomes 6 and 18, and only one *PtrIBI* gene on the remaining six chromosomes. A cluster of *PtrIBI* duplicated genes (*PtrIBI11/PtrIBI12*) was found on chromosome 18.

Next, we analyzed the physicochemical properties of the proteins in the entire *PtrIBI* genes family. The *PtrIBI* genes generally encode 545 to 703 amino acids, with an average of 604.5 amino acids. The molecular weights of the *PtrIBI* proteins are relatively large, all being greater than 60 kDa. The theoretical pI of *PtrIBIs* ranges from 5.64 to 8.26, with 10 genes encoding acidic proteins and 2 genes encoding basic proteins. Four members of the *PtrIBI* gene family have instability index values less than 40 and are considered stable, while the remaining proteins are unstable. All the *PtrIBI* family proteins are hydrophilic (Supplementary Table S2). The Plant-mPLoc database predicts the majority of the *PtrIBI* proteins to be located in the cytoplasm.

### Evolutionary relationship and sequence analysis of *PtrIBIs*

**Fig. 1:**
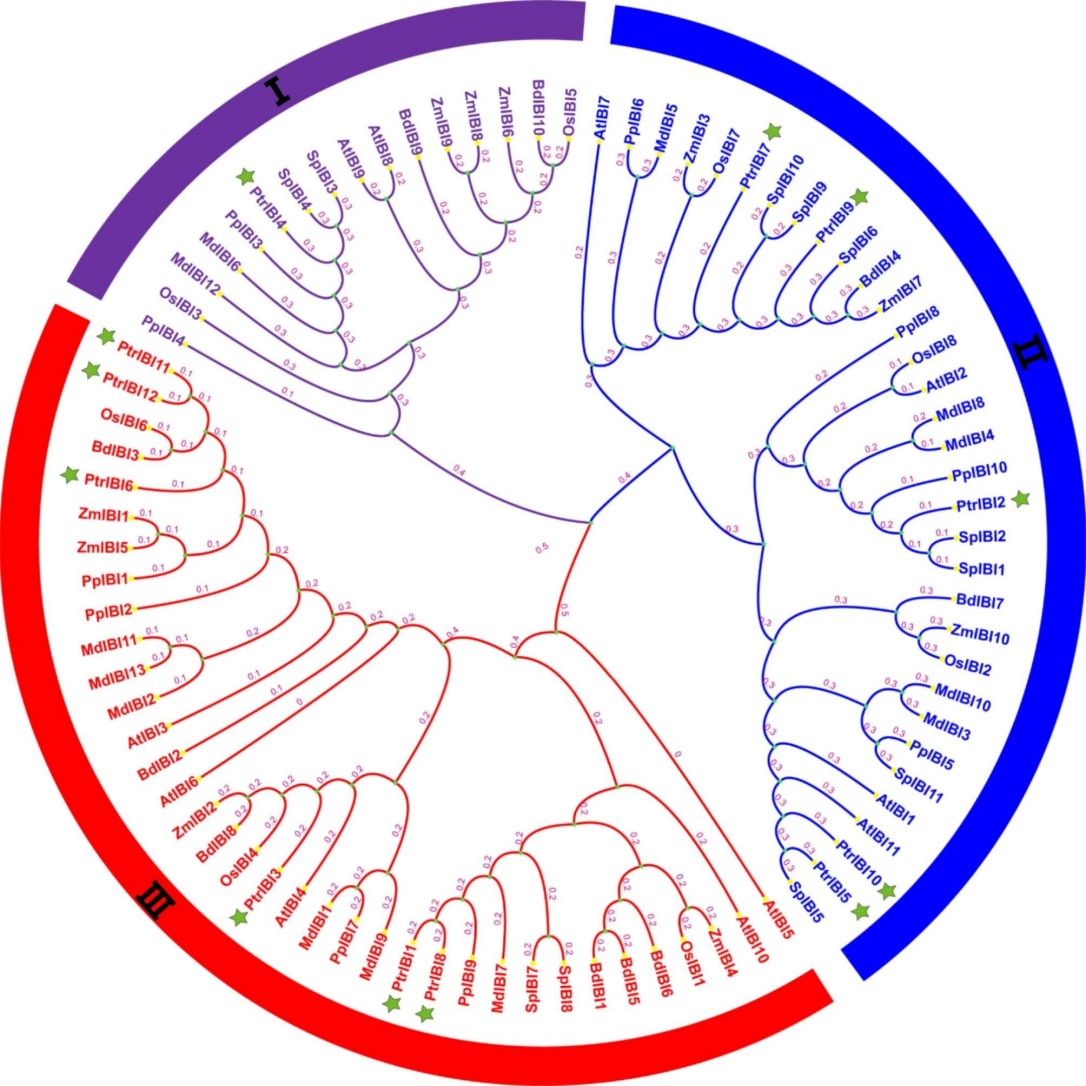
Evolution and phylogenetic analysis of the *IBI* family among different plants. The genealogical tree of Aspartyl tRNA-synthetase from *Populus trichocarpa*, *Salix purpurea*, *Arabidopsis thaliana*, *Oryza sativa*, *Malus domestica*, *Zea mays*, *Prunus persica*, *Brachypodium distachyon*

We used the amino acid sequences of eight plant AspRS to build a biogenetic tree to further investigate the evolution and differences of plant AspRS family proteins (Fig. 1). Phylogenetic tree analysis showed that there were five *PtrIBI* genes on branch two and six *PtrIBI* genes on branch three, except for *PtrIBI4* on branch one. Some of the *PtrIBI* genes have high homology (e.g., *PtrIBI1/PtrIBI8, PtrIBI5/PtrIBI10, PtrIBI11/PtrIBI12*). We selected the AspRS amino acid sequences of five species and performed sequence comparison using CLUSTALX (Fig. S3). The results showed that they are somewhat conserved. In general, the AspRS gene family is highly conserved evolutionarily. The analysis revealed that the protein tertiary structures of *PtrIBI4* (*P. trichocarpa*) and *AtIBI1* (*A. thaliana*) were very similar.

### Gene family motifs, conserved structural domains and gene structure analysis of *PtrIBIs* in *P. trichocarpa*

To gain further insight into the evolutionary relationships among various members of the *PtrIBI* genes family, we constructed an evolutionary tree (Fig. S4a) and investigated their conserved protein motifs, structural domains, and gene structures (Fig. S4b-d). Our analysis revealed 10 conserved motifs (Fig. S4b) (Supplementary Table S3) and 7 conserved structural domains (Fig. S4c) in the *PtrIBI* protein sequence. Notably, all *PtrIBI* genes share motif 5/3/1, and the most conserved structural domain among *PtrIBIs* is PLN02502 (aminoacyl-tRNA ligase). Additionally, all *PtrIBI* genes contain introns and UTRs, with the number of introns ranging from 5 to 16 (Fig. S4d). Specifically, *PtrIBI11* and *PtrIBI2* have a maximum of 16 introns, while *PtrIBI5* and *PtrIBI10* have a minimum of five introns. Accordingly, the *PtrIBI* genes on each branch of the evolutionary tree exhibit a highly similar conserved motif arrangement.

### Collinearity Analysis of *PtrIBI* Genes

**Fig. 2:**
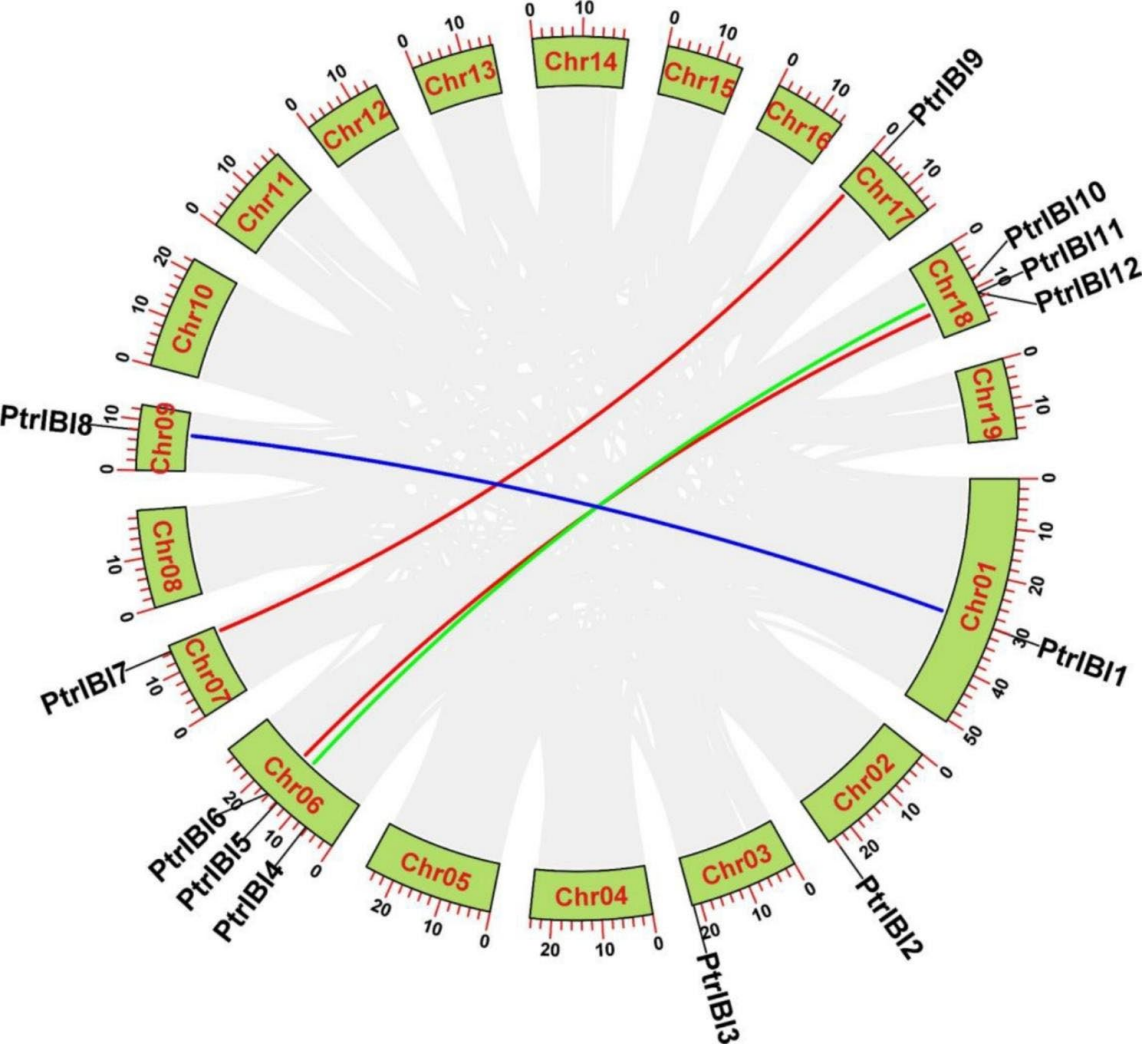
Evolutionary relationship analysis of the *PtrIBI* genes family. Evolutionary analysis of the *PtrIBI* genes family in *P. trichocarpa*, with different sizes of fan-shaped rings represent different sizes of chromosomes. The gray and colourful connecting genes show all collinearity blocks and the fragment doubling event

**Fig. 3:**
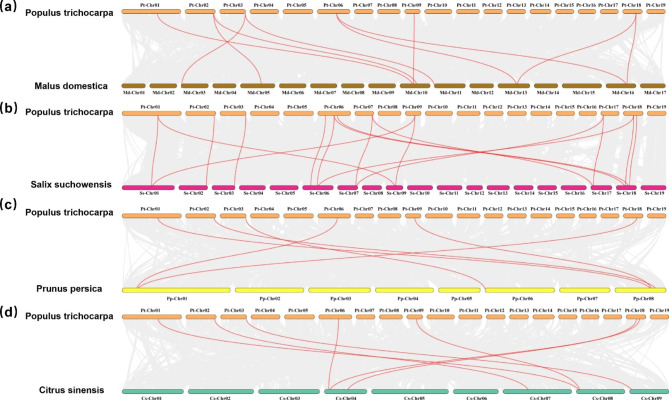
Collinear analysis of *PtrIBI* genes in *P. trichocarpa* with 4 other plants. Gray lines in the background represent collinear blocks of *P. trichocarpa* and other species genomes, while red lines emphasize collinear *PtrIBI* genes pairs. (**a**) *Malus domestica*. (**b**) *Salix suchowensis*. (**c**) *Prunus persica*. (**d**) *Citrus sinensis*

During evolution, *PtrIBIs* have undergone multiple duplication events. The genes *PtrIBI1* and *PtrIBI8*, *PtrIBI6* and *PtrIBI11*, *PtrIBI5* and *PtrIBI10*, *PtrIBI7* and *PtrIBI9* are whole genome duplication (WGD) pairs, as demonstrated by Fig. 2 and Supplementary Table S4, indicating that they share a common ancestor. The Ka/Ks values of all four groups of genes were less than 1, implying that these genes underwent purifying selection, as shown in Supplementary Table S5. We then created comparative collinear graphs of the *PtrIBI* gene family in *P. trichocarpa* with eight woody plants, such as *Malus domestica*, *Salix suchowensis*, *Prunus persica*, *Citrus sinensis*, *Ziziphus jujuba*, *Theobroma cacao*, *Vitis vinifera*, and *Punica granatum* (Fig. 3) (Fig. S5a-d). The collinear graphs indicate that *P. trichocarpa* has 10 pairs of AspRS homologs with *Malus domestica*, 17 pairs of AspRS homologs with *Salix suchowensis*, six pairs of AspRS homologs with *Prunus persica*, seven pairs of AspRS homologs with *Citrus sinensis*, three pairs of AspRS homologs with *Ziziphus jujuba*, *Theobroma cacao*, and *Vitis vinifera*. Moreover, *Ziziphus jujuba*, *Theobroma cacao*, *Vitis vinifera*, and *Punica granatum* have 5 and 3 pairs of AspRS homologs, respectively. We also created comparative collinear graphs of the *PtrIBI* gene family in *P. trichocarpa* with four herbaceous plants, including *Arabidopsis thaliana*, *Oryza sativa*, *Triticum aestivum*, and *Zea mays*, demonstrating that *P. trichocarpa* has five pairs of AspRS homologs with *Arabidopsis thaliana*, but no AspRS homologs with *Oryza sativa*, *Triticum aestivum*, and Z*ea mays* (Fig. S5e-h).

### *PtrIBIs* cis-element analysis

**Fig. 4:**
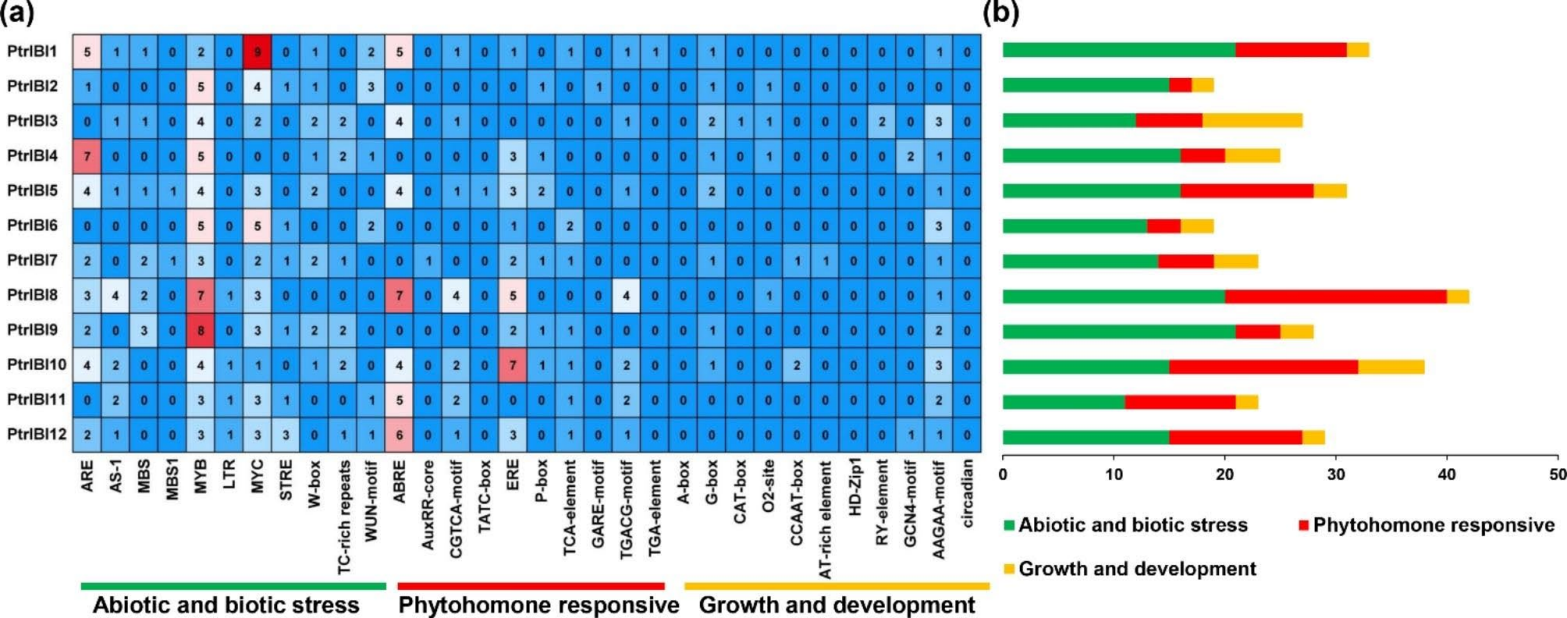
The cis-acting element of the *PtrIBI* genes. (**a**) Numbers and gradient red indicate the number of cis-acting elements; (**b**) color-coded histograms indicate the number of cis-acting elements for each type of gene, which are divided into three categories by functional factors: phytohormone responsive, abiotic and biotic stress, plant growth and development

To determine the expression pattern of *PtrIBI* genes family, the cis-elements of the *PtrIBIs* promoter were analyzed using the PlantCARE database (Fig. 4). These elements are involved in abiotic and biotic stresses, plant hormone responses and plant growth and development. We visualized the promoter elements at the promoter positions (Fig. S6). The specific functions of these cis-elements are labeled (Supplemental Table S6). Stress-related cis-elements (Myb, Myc, ARE) were enriched in some genes, suggesting that these *PtrIBI* genes may play a key role in response to adverse conditions. In addition, some *PtrIBI* genes promoters were enriched for ABRE (involved in ABA response), such as *PtrIBI8/PtrIBI12*, and these genes may be responsive to ABA hormone. Promoters of *PtrIBI8/10/11/12* contained LTR elements (involved in low temperature stress response), suggesting that these genes may be responsive to low temperature induction.

### Transcriptome analysis of *PtrIBI* genes in *P. trichocarpa*

**Fig. 5:**
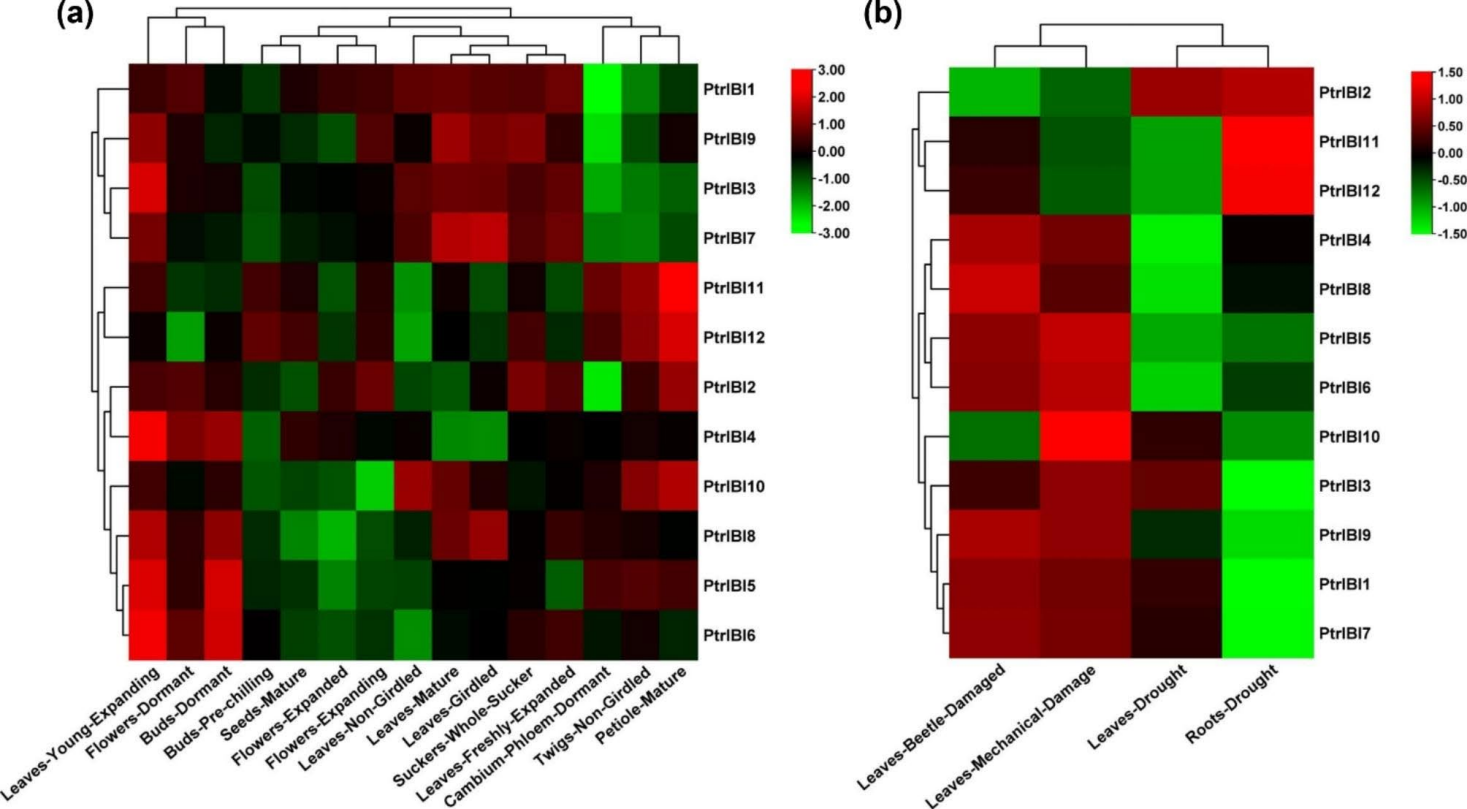
Expression profiles of *PtrIBI* genes under developmental and stress conditions. (**a**). The expression levels of *PtrIBI* genes in different tissues at different developmental stages are plotted based on transcriptome data. (**b**). Depicts a heat map of gene expression levels of *PtrIBIs* following drought, beetle and mechanical injury. The color bar represents the range of maximum and minimum values for relative expressions in the heatmap

To further investigate the role of *PtrIBI* genes in growth, development, and stress, we downloaded tissue expression profiles and stress-induced expression profiles of the *PtrIBI* gene family from the transcriptome database (Supplemental Table S7, S8). We generated a heat map depicting the clustering of samples and genes in two directions to examine the expression patterns of *PtrIBI* genes across 15 poplar tissues (Fig. 5a). The majority of these genes exhibited high expression levels in young shoots, such as *PtrIBI4/5/6*. Some genes, such as *PtrIBI4/6*, were highly expressed in young and expanding leaves, which may be associated with the growth and development of poplar. To further investigate their response to stress, we examined the expression of the *PtrIBI* genes family under conditions of drought, beetle infestation, and mechanical damage (Fig. 5b). Most of the genes in the *PtrIBI* genes family were up-regulated under beetle and mechanical damage, suggesting that these genes may be responsive to leaf damage stress. Some genes were up-regulated under drought stress (e.g., *PtrIBI11/12*), and they may play a role in drought stress.

We next subjected poplar to drought and different concentrations of BABA treatment under drought. The results showed that after the addition of 50 mM BABA at the drought treatment, poplar leaves were greener and less wilted than those of the drought treatment and 200 mM BABA drought treatment (Fig. S7a). We measured relative electrical conductance (REC), chlorophyll content, leaf relative water content (leaf RWC), and Fv/Fm (maximal PSII quantum yield) under different treatments. The results indicated that the addition of 50 mM BABA at drought treatment would alleviate the damage of drought stress (Fig. S7b-e).

### Expression pattern and analysis of *PtrIBI* genes in poplar under drought treatment

**Fig. 6:**
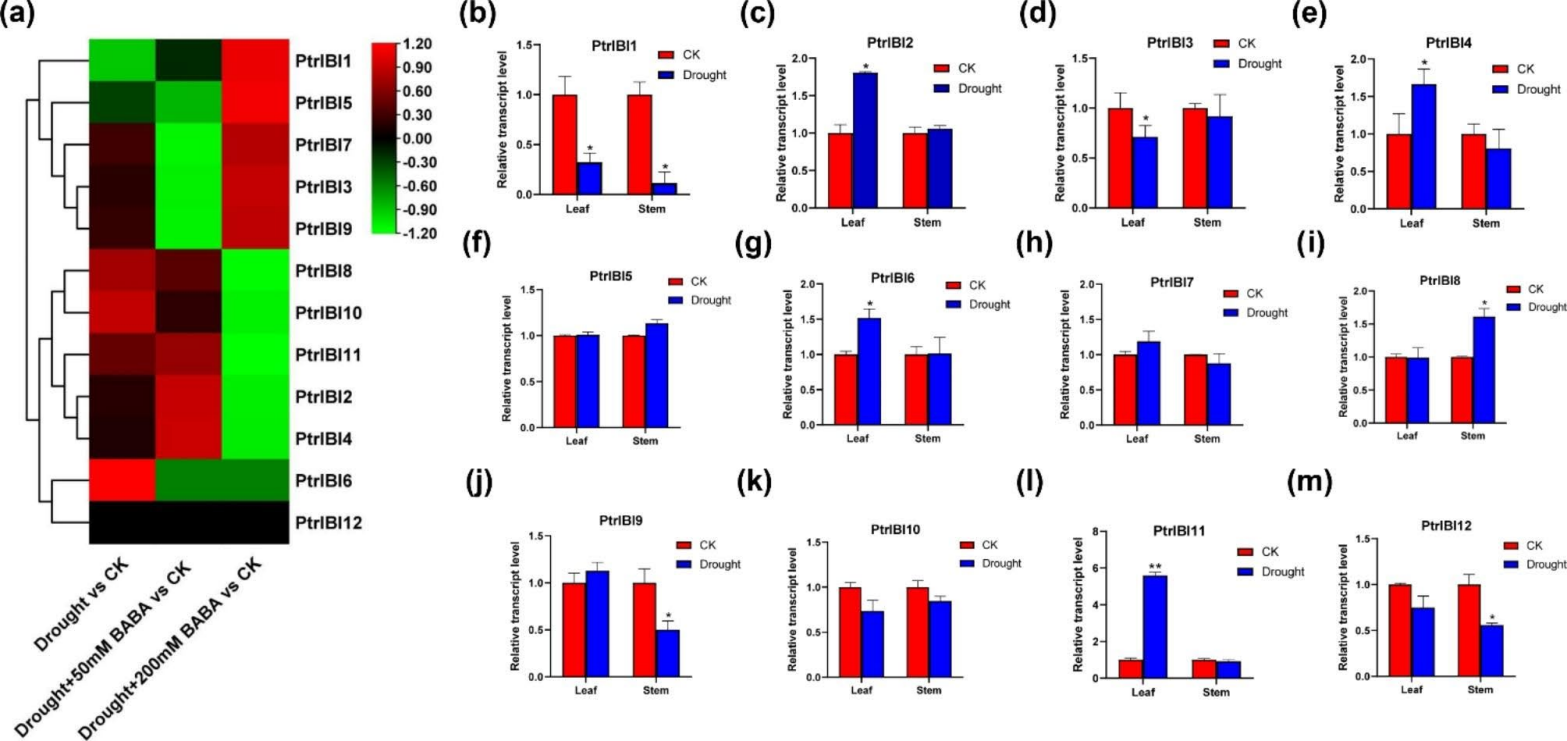
Transcriptome and qRT-PCR analysis of *PtrIBI* genes expression under drought stress in *P. tomentosa*. (**a**) RNA-seq analysis of 12 *PtrIBI* genes under drought, drought + 50 mM BABA and drought + 200 mM BABA. (**b**-**m**) Transcriptional levels of the 12 *PtrIBI* genes in response to drought stress in the leaves and stem of *P. tomentosa*

*PtrIBI* genes encode AspRS proteins, the receptors of BABA, which play an important role in ABA signaling-mediated drought stress. RNA-seq and qRT-PCR were performed to analyze the transcript levels of *PtrIBI* genes under drought and different concentrations of BABA treatment to verify whether the poplar *PtrIBIs* family can respond to drought stress. Transcriptome data showed that the expression levels of *PtrIBI6/8/10/11* genes were elevated in leaves under drought treatment (Fig. 6a) (Supplemental Table S9). The expression levels of *PtrIBI2/4/11* genes were significantly triggered in the leaves under 50 mM BABA drought treatment. The expression of *PtrIBI1/3/5/7/9* genes was elevated under 200 mM BABA drought treatment. The expression levels of 12 *PtrIBI* genes under normal conditions and drought stress were examined by qRT-PCR to verify the expression of these genes in the transcriptome (Fig. 6b-m). The experimental results showed that *PtrIBI2/4/6/11* were highly expressed in drought-treated leaves and *PtrIBI8* were highly expressed in drought-treated stems. The results indicated that the poplar *PtrIBI* genes family responded differently to drought treatment in different tissues.

### Molecular network of *PtrIBI* genes regulating drought stress in *P. trichocarpa*

**Fig. 7:**
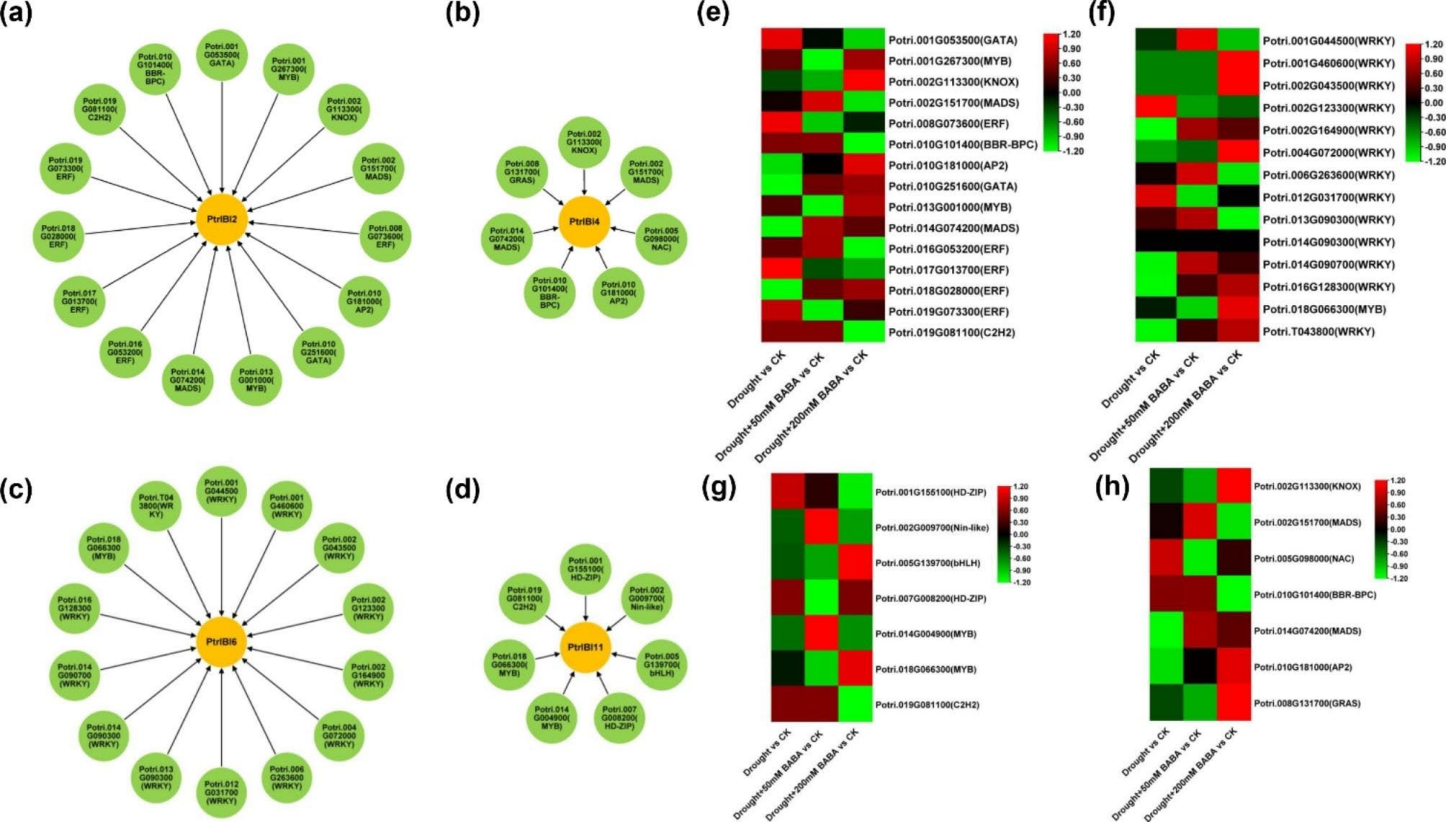
Bioinformatic analysis of transcription factors (TFs) of *PtrIBIs* in poplar. (**a**-**d**) TFs-*PtrIBI2/4/6/11* interaction network analysis. Green circles indicate TFs upstream of *PtrIBIs*, and yellow circles represent *PtrIBI2/4/6/11*. Heat map showing the transcriptional abundance of *PtrIBI2*(**e**), *PtrIBI6* (**f**), *PtrIBI11* (**g**) and *PtrIBI4* (**h**) upstream transcription factors under drought, drought + 50 mM BABA and drought + 200 mM BABA. The range of fold change in expression in the heat map is indicated by the colour bar

Upstream TFs of *PtrIBIs* were identified using bioinformatics to explore the regulatory network of *PtrIBIs* under drought stress (Supplemental Table S10). The *PtrIBIs* interaction network showed that the expression of *PtrIBI2/4/6/11* was regulated by 15, 7, 14 and 7 TFs (Fig. 7a-d), and *PtrIBI3/5/8/9/10/12* was regulated by 7, 10, 8, 14, 5 and 27 TFs (Fig. S8). These transcription factors include NAC, MYB, BES1, BBR-BPC, Dof, ERF, HD-ZIP, Nin-like, AP2, TCP, LBD, GATA, GAGA, KNOX, C2H2, and MADS family proteins. In addition, the same transcription factor may simultaneously affect the expression of different *PtrIBI*. Potri.014G074200(M_MADS) may simultaneously affect *PtrIBI2/3/4/5/9*.

**Fig. 8:**
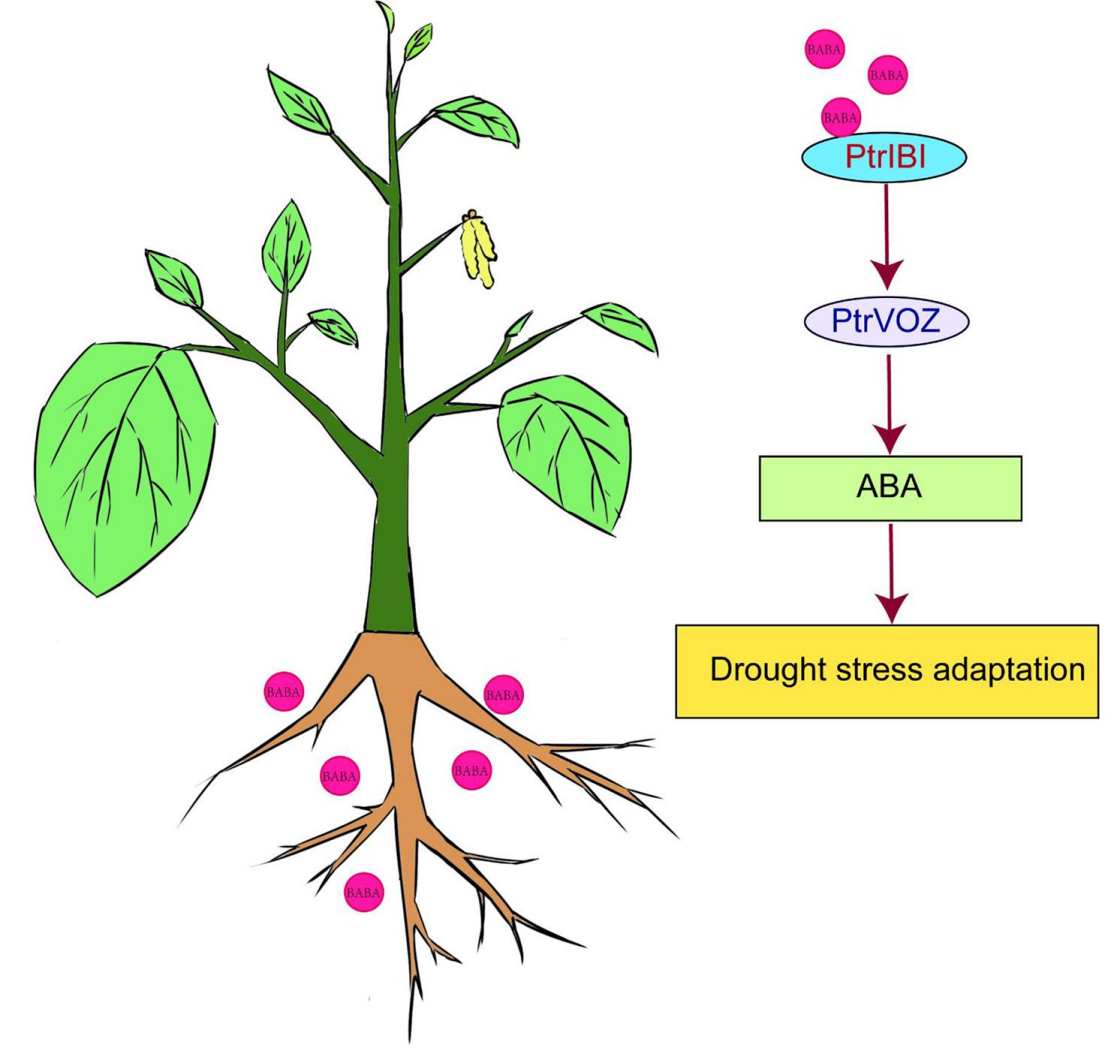
Schematic model of the response of *P. tomentosa PtrIBI* genes family under drought stress

The transcription levels of *PtrIBI2/4/6/11* upstream TFs in poplar leaves under drought treatment and different concentrations of BABA drought treatment were shown in figure (Fig. 7e-h). Under 50 mM BABA drought treatment, the upstream TF Potri.002G151700 (MIKC_MADS ) of *PtrIBI2* was significantly upregulated (Fig. 7e), and the expression of Potri.014G074200 (M_MADS) and Potri.016G053200 (ERF) was slightly upregulated. Potri.002G151700 (MIKC_MADS ), Potri.010G101400 (BBR-BPC), and Potri.014G074200 (M_MADS) expressions in *PtrIBI4* upstream TF were also upregulated (Fig. 7h). Similarly, Potri.001G044500 (WRKY), Potri.006G263600 (WRKY) were significantly up-regulated in *PtrIBI6* upstream TF under 50 mM BABA and drought treatment (Fig. 8f), and Potri.002G009700 (Nin-like), Potri.014G004900 (MYB) were significantly up-regulated expression in *PtrIBI11* upstream TF (Fig. 7g).

### Expression analysis of key genes in ABA pathway under different treatments

*AtVOZ* are chaperones of AspRS protein interactions, and their transcription is induced by ABA. Our analysis of ABA signaling pathway transduction showed that exogenous application of BABA resulted in increased *PtrVOZ* gene expression. We found that exogenous application of BABA resulted in uneven distribution of pyrabactin resistance/pyrabactin resistance-like (*PYR/PYL*) gene expression in stems and leaves, with Potri.001G142500 (*PtrPYR1*), Potri.014 G097100 (*PtrPYL10*) were highly expressed in leaves, and Potri.001G092500 (*PtrPYL1*), Potri.003G139200 (*PtrPYL5*) were highly expressed in stems. Most *PP2Cs* genes were up-regulated in expression in stems and leaves under 200 mM BABA drought treatment. Potri.008G059200 (*PtrPP2C*) was highly expressed in leaves and Potri.015G018800 (*PtrPP2C*) was highly expressed in stems under 50 mM BABA drought treatment. Under 50 mM BABA drought treatment, Potri.004G218200 (*PtrSnRK2*) was expressed up-regulated in leaves and down-regulated in stems. Under 200 mM BABA drought treatment, *PtrSnRK2* was expressed down-regulated in leaves and significantly up-regulated in stems. Potri.001G404100 (*PtrRD26*), Potri.014G0908009 (*PtrLEA14*) are important genes in the ABA pathway associated with drought stress. They are highly expressed in leaves under BABA-treated drought stress. *PtrRD26* is also highly expressed in stems, and *PtrLEA14* is down-regulatedly expressed in stems.

## Discussion

β-aminobutyric acid (BABA) is a priming agent that provides broad-spectrum disease protection [20]. Aspartyl tRNA-synthetase (AspRS) family genes can encode cytoplasmic proteins that specifically bind intracellularly to BABA, which sends atypical defense signals in the cytoplasm after pathogen attack [38, 40]. At present, the AspRS family has been defined and mined in a variety of plants including Arabidopsis, rice, and tomato [61–63]. However, its distribution and function in poplar have not been studied. We identified AspRS family genes of poplar using comparative genomics and transcriptomics approaches and analyzed their responses to abiotic stresses. We next used bioinformatics to analyze the transcriptional regulatory network of poplar under drought treatment, which provides a molecular basis for poplar response to drought stress [3, 5, 8, 53, 67]. Transcriptome data showed that the expression levels of *PtrIBI6/8/10* genes were significantly triggered in leaves under drought treatment (Fig. 6a) and *PtrIBI2/4/11* genes were significantly elevated in leaves under 50 mM BABA drought treatment.

### Identification and evolutionary analysis of the *PtrIBIs* family in *P. trichocarpa*

We detected 12 *PtrIBI* genes in *P. trichocarpa* and characterized their phylogenetic tree and expression profile. These 12 *PtrIBI* genes were distributed on chromosomes (Chr1, 2, 3, 6, 7, 9, 17, and 18) and were named *PtrIBI1* to *PtrIBI12* according to their positions (Supplemental Table S1 and Fig. S2). Since these 12 genes encode the Aspartyl tRNA-synthetase (AspRS) protein family, their amino acid sequences are highly hydrophilic (Supplemental Table S2). The AspRS proteins are mainly located in the cytoplasm and have one conserved structural domain (Fig. S4b, c). The conserved structural domain ensures that BABA may bind to AspRS proteins and thus cause plants to respond to external environmental stresses. Multiple introns exist in poplar *PtrIBI* genes (Fig. S4d). It has been shown that genes lacking introns are more likely to complete the transcription process and form mRNA. So *PtrIBI* genes may take time to develop a response under stress.

These findings suggest that AspRS share an anti-parallel beta-sheet fold flanked by alpha-helices [63]. We constructed a genealogy tree based on eight different plant protein sequences and performed amino acid sequence comparison for five of them. The results revealed that different plant IBI genes have high homology, and their amino acid sequence comparisons indicate that they are evolutionarily conserved. In addition, phylogenetic analysis of family genes can explain the evolution of genes. Poplar and willow belong to the same genus, so their *IBI* genes are relatively clustered.

The mechanisms of gene family membership increase and genome evolution are largely dependent on gene duplication events, including whole genome duplication (WGD) and tandem duplication (TD) [64]. In the study, 12 *PtrIBI* genes were distributed on eight chromosomes with four WGD gene pairs and one TD gene pair. Tandem duplicated genes may have similar functions and expression patterns (Fig. 5a-b). Significantly, *PtrIBI7/9* with high homology formed gene pairs through WGD, and they had similar transcript levels in different tissues and under different stresses (Fig. 5a-b).

### Potential functional analysis of the poplar *PtrIBI* genes family

Although the function of the AspRS protein family has been characterized in many species, it has changed with the evolution of different species. The transcript levels of genes are important to assess the function of genes. We analyzed poplar transcriptome data, the expression of *PtrIBI* genes fluctuated slightly in each poplar tissue. *PtrIBI4/5/6* were highly expressed at young shoots, which indicated that some of the *PtrIBI* genes might be associated with the growth and development of poplar.

AspRS protein acts as a receptor protein for BABA, which triggers defense responses controlled by salicylic acid (SA)-dependent and non-dependent signaling pathways [19, 65]. Transcriptome datasets showed that most *PtrIBI* genes were highly expressed under leaves beetle damaged and leaves mechanical damage stresses. *PtrIBI2/11/12* were significantly up-regulated in expression under drought stress, while *PtrIBI1/3/4/7/9* were down-regulated under drought stress. It was found that the transcript levels of *PtrIBI* genes were not consistent under biotic and abiotic stresses. The transcriptional regulation of genes depends largely on their promoters, so we performed promoter analysis of the *PtrIBI* genes family. The results indicate a large number of abiotic and biotic stress initiation elements on the promoters of the *PtrIBI* genes family, especially ABRE and ERE. *PtrIBI1/8/11/12* all possess five and more ABRE elements on their promoters, and *PtrIBI8/PtrIBI10* possess five and more ERE elements on their promoters. In addition, ABRE and ERE elements play an important role in response to abiotic stresses [51, 57]. In conclusion, plants can respond to various stresses and adapt to complex external environments by regulating the expression levels of *PtrIBI* genes.

### Molecular regulatory networks of *PtrIBIs* involved in drought stress

Previous studies have reported that AspRS proteins in plants such as *Arabidopsis*, rice, and tobacco can bind specifically to BABA and thus coordinate downstream signaling pathways to resist stress. In *Arabidopsis*, *AtIBI1* binds to *AtVOZ* and affects the ABA signaling pathway, playing an important role in resisting cold stress [38]. The ABA signaling pathway in poplar is activated in response to drought stress. Poplar enhances its drought resistance by closing stomata and increasing peroxide-scavenging enzymes. Therefore, it is crucial to explore how poplar can regulate the ABA signaling pathway to improve its drought resistance [3]. We subjected poplars to drought treatment and external application of different concentrations of BABA drought treatment, and found that external application of low concentration of BABA could make poplars resist drought. Poplar leaves showed wilting and yellowing after drought treatment, and chlorophyll content and water use efficiency were reduced. Topical application of 50 mM BABA resulted in lower relative electrical conductance, indicating less damage to poplar cell membranes. However, maximal PSII quantum yield was higher and poplar trees had higher photosynthetic capacity. It was shown by RNA-seq data that exogenous application of BABA during drought affects the transcript levels of *PtrIBI* genes. *PtrIBI2/4/11* were highly expressed upon external application of 50 mM BABA drought treatment, and they may play an important role in external application of BABA to resist drought stress. The qRT-PCR results revealed that the *PtrIBI* genes family also had inconsistent transcript levels in the stem and leaf spaces after drought treatment. Notably, *PtrIBI1* expression was decreased in both stems and leaves after drought treatment.

A recent study has shown that the *IBI1*-*VOZ* signaling module can transduce ABA signaling. However, there are few reports on how *PtrIBI* genes function through transcriptional regulation. Transcriptional regulation plays a crucial role in all aspects of the plant life cycle and transcription factors play a central role in transcription. Therefore, it is crucial to identify the upstream transcription factors of *PtrIBI* genes. We therefore used bioinformatic methods to identify upstream transcription factors of the *PtrIBI* genes, and we found that some potential upstream transcription factors of *PtrIBIs* were significantly up-regulated in drought-treated leaves. MYB, WRKY and ERF TFs can bind to elements (e.g., MYB and AREB) on the promoters of downstream genes in many plants (e.g., Arabidopsis and poplar). For example, the poplar ERF TF *ERF16* exerts salt tolerance by binding to the promoter of *NAC45* (containing the ERF element) [66]. *WRKY77* negatively regulates plant tolerance to salt stress by binding to the *RD26* and *NAC002* promoters [16, 17].

**Fig. 9:**
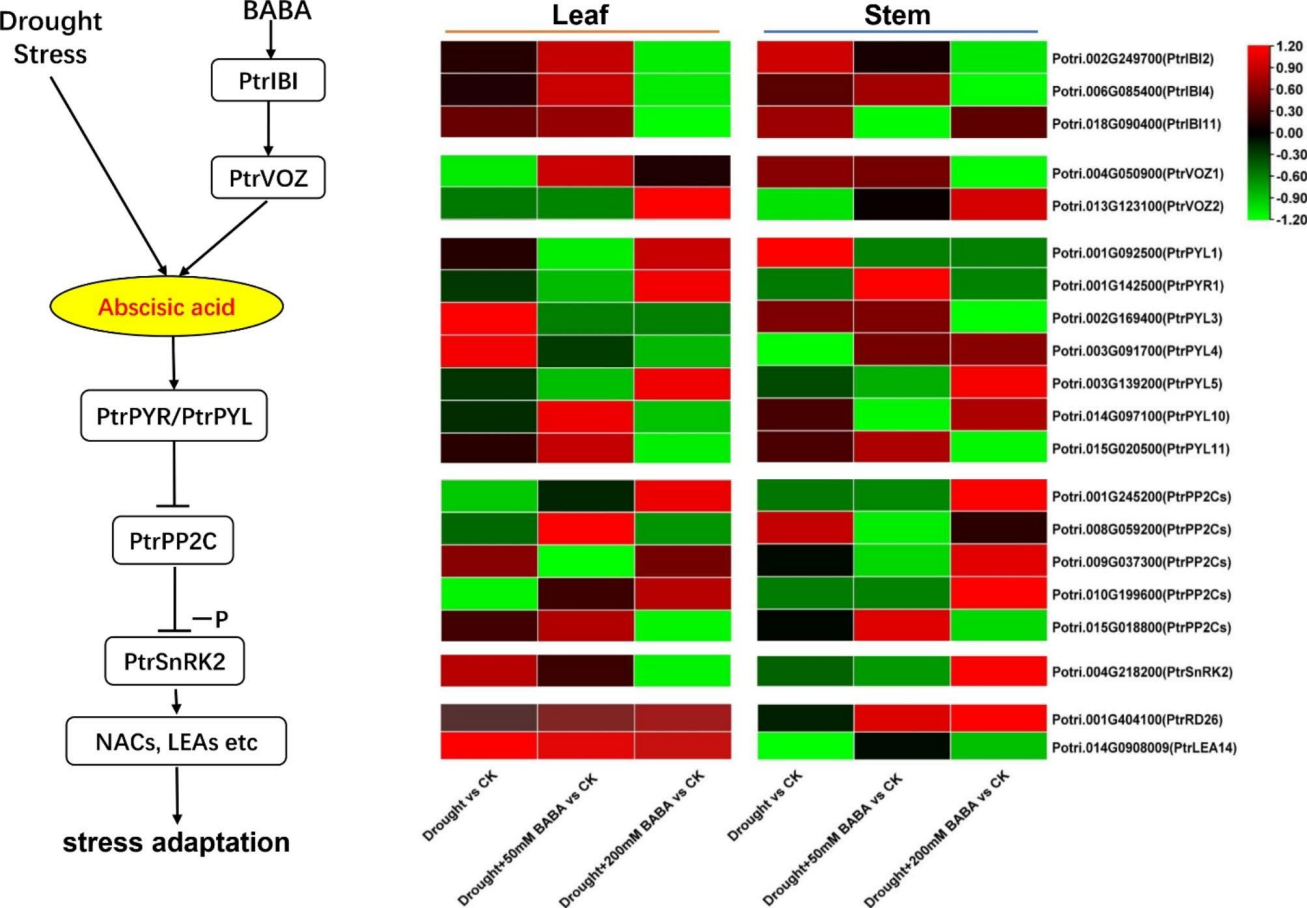
The *PtrIBI* genes mediates the abscisic acid signaling pathway in *P. tomentosa*. Poplars were divided into four treatment groups: CK, drought, drought + 50 mM BABA and drought + 200 mM BABA; each treatment was applied for 15d. Heatmaps represent the log^2^(FC) of genes under different treatments. The boxes in the pathway indicate DEGs. Green and red indicate downregulated and upregulated genes, respectively

Thus, several TFs (e.g., ERF transcription factors and MYB transcription factors) may bind to elements such as ERF and MYB in the promoters of *PtrIBI* genes, thereby regulating the expression of *PtrIBI* genes. In conclusion, the TF-*PtrIBIs* module plays a crucial role in the regulation of plant responses to drought stress. To verify that the BABA-*PtrIBIs*-*PtrVOZ* signaling module can conduct ABA signaling in poplar, we analysed the expression of key genes in the ABA pathway in various treatments using transcriptomic data. It was found that genes on the ABA signaling pathway were transcribed at different levels in the stem and leaves, and that the same gene family was transcribed at different levels under the same treatment (Fig. 9). Transcriptome results revealed that both the drought-related genes *PtrRD26* and *PteLEA14* were highly expressed in the leaves. *PtrRD26* was highly expressed in BABA-treated stems and may be associated with resistance to drought stress in poplar after 50 mM BABA treatment. In conclusion, the BABA-*PtrIBIs*-*PtrVOZ* signaling module may play an important role in ABA signaling in poplar, thereby affecting drought resistance in poplar [67].

## Conclusion

Based on the experimental data, we constructed a model map of the response of the *PtrIBI* genes family poplar to drought stress (Fig. 8). In summary, genome-wide analysis of the Aspartyl tRNA-synthetase (AspRS) family of *P. trichocarpa* identified 12 *PtrIBI* genes. Genomics and bioinformatics were used to determine the chromosomal localization, evolutionary tree, gene structure, gene doubling, promoter cis-elements and expression profiles of *PtrIBIs*. We found that some *PtrIBI* genes can be significantly regulated by drought, beetle and mechanical damage, suggesting that *PtrIBIs* play an important role in poplar stress tolerance. Finally, external application of low concentrations of BABA increased plant drought resistance under drought stress. Plants can transduce ABA signaling in poplar through the BABA-*PtrIBIs*-*PtrVOZ* signaling module, and the module regulates their response to drought stress. The results of this study allowed us to predict the possible characteristics of the *PtrIBI* genes in poplar and suggest that poplar can be improved for drought tolerance with topical application of low concentrations of BABA.

### Electronic Supplementary Material

Below is the link to the electronic supplementary material.

Supplementary Material 1

Supplementary Material 2

## Data Availability

All data supporting the conclusions of this paper are provided in the article and its additional files. Genome sequence data for all species are available in the Phytozome database (https://phytozome.jgi.doe.gov/pz/portal.html). Publicly available RNA-seq data are available on PopGenIE (https://popgenie.org/).

## Abbreviations

AspRS: Aspartyl tRNA-synthetase
BABA: β-aminobutyric acid
WGD: Whole genome duplication
CDS: Coding sequence
AA: Amino acid
MW: Molecular mass (kDa)
ROS: Reactive oxygen species
ABA: Abscisic acid
PYL: Pyr1-like proteins
TFs: Transcription factors
REC: Relative electrical conductance. ERF: Ethylene Response Elements
BBR-BPC: Barley B RECOMBINANT/BASIC PENTACYSTEINE
NAC: NAM、ATAF1/2、CUC1/2 gene family
BES1: BRI1-EMS-SUPPRESSOR 1
bHLH: Basic/helix-loop-helix
MYB: MYB DNA-binding domain
MIKC_MADS: MIKC-type MADS
M_MADS: M-type MADS
AP2: Ethylene Response Elements
C2H2: C2H2 zinc-finger
HD-ZIP: HD(Homeodomain) domain and Leu zipper(Zip) component
GRAS: GAI, RGA, SCR gene family
Dof: DNA Binding with one finger
Nin-like: NIN-Like Protein
TCP: TEOSINTE BRANCHED 1(TB1)、
CYCLOIDEA(CYC)和: PROLIFERATING CELL FACTORS(PCFs)
LBD: Lateral Organ Boundaries Domain
GAGA: GAGA-binding transcription factor
KNOX: KNOTTED1 like homeobox
*AtIBI*: Aspartyl tRNA-synthetase of *Arabidiopsis thaliana*
*AtVOZ*: Vascular plant one zinc-finger of *Arabidiopsis thaliana*
