# Supplementary material for: Aspartyl tRNA-synthetase (AspRS) gene family enhances drought tolerance in poplar through BABA-PtrIBIs-PtrVOZ signaling module
Source: BMC Genomics. 2023 Aug 21;24:473. doi: 10.1186/s12864-023-09556-2 (PMC10441740; doi:10.1186/s12864-023-09556-2)
Supplement: Supplementary file 2 — Supplementary Material 2 [file 12864_2023_9556_MOESM2_ESM.zip › 12864_2023_9556_MOESM12_ESM.docx]

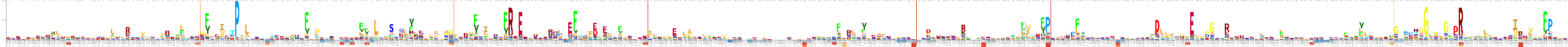


**Fig. S1.** Aspartyl tRNA-synthetase motif (PF00152) sequencing of all proteins in *P. trichocarpa*. Numbers on the x-axis represent sequence positions in the Aspartyl tRNA-synthetase pattern. The numbers on the y-axis represent information content measured in bits. Serial logos are generated using the WebLogo tool.


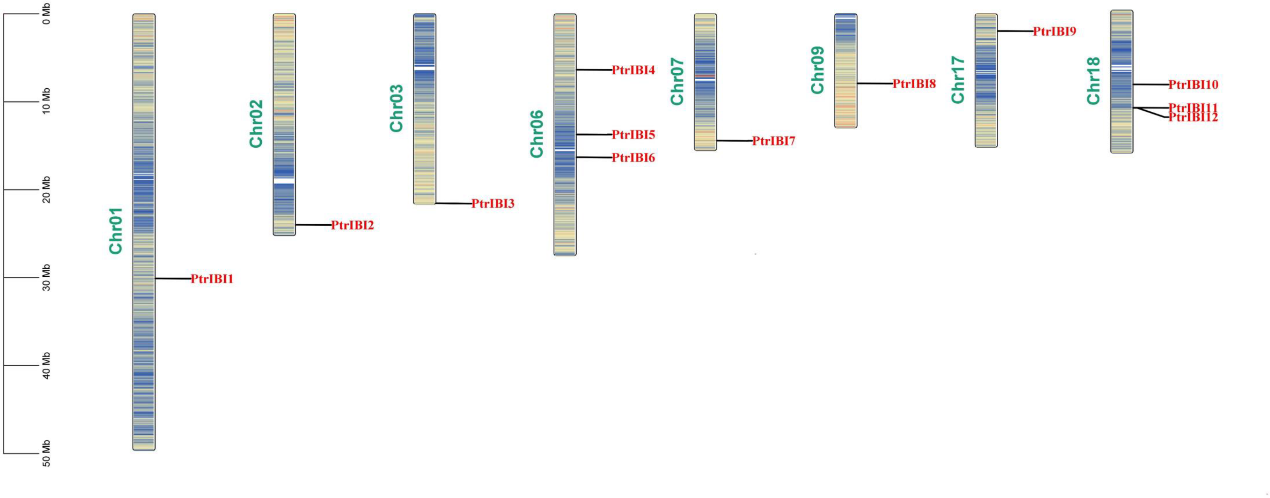


**Fig. S2.** Localization of 12 PtrIBIs on P. trichocarpa chromosomes. The scale on the left is in megabytes. The serial number of the chromosome is shown to the left of each. The gene names to the right of each chromosome correspond to the approximate location of each PtrIBIs gene. Chromosome colors represent gene abundance.


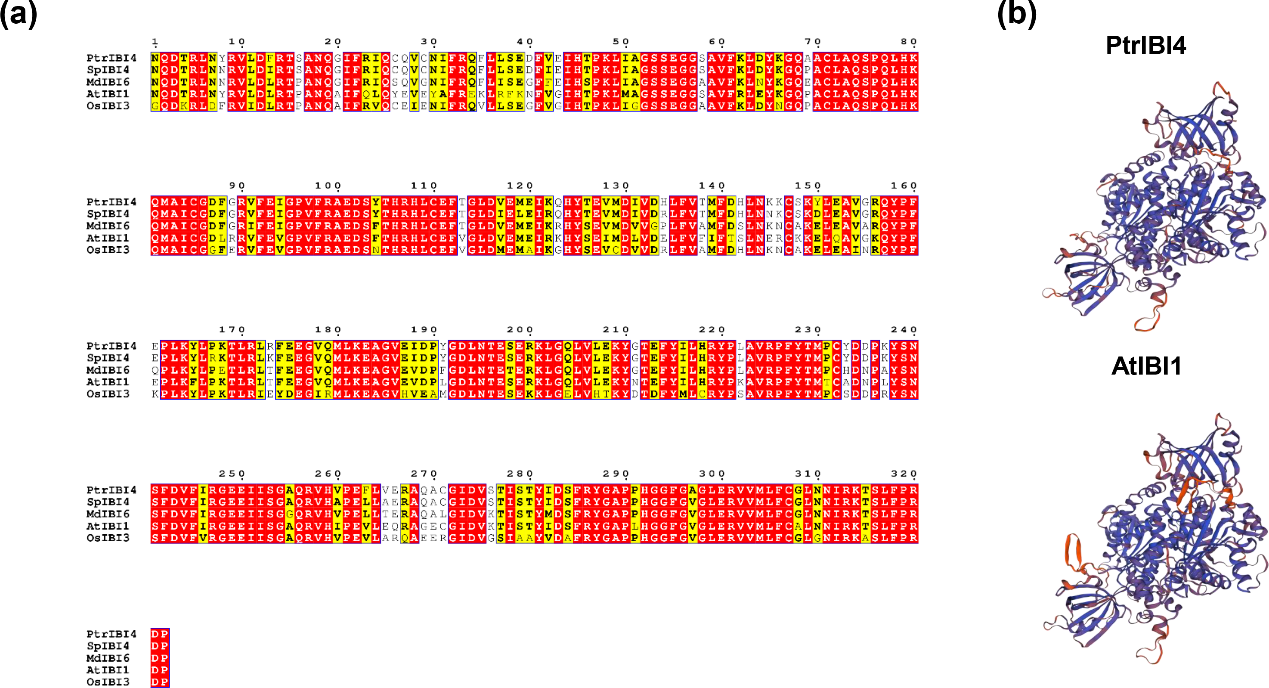


**Fig. S3.** Representative PtrIBIs protein sequence alignment of each species. (a) Using CLUSTALX to compare *PtrIBI4* (*P. trichocarpa*), *SpIBI4* (*S.purpurea*), *MdIBI6* (*M. domestica*), *AtIBI1* (*A. thaliana*), *OsIBI3* (*O.sativa*). (b) Predicted protein tertiary structure of *PtrIBI4* (*P. trichocarpa*) and *AtIBI1* (*A. thaliana*).


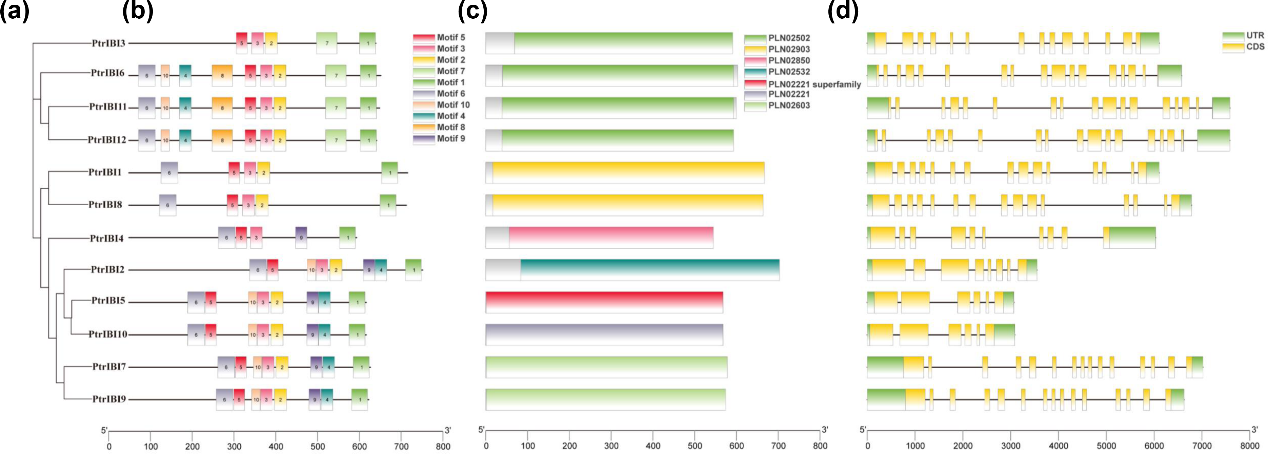


**Fig. S4.** Gene family motifs, conserved structural domains and gene structure analysis of PtrIBIs in *P. trichocarpa*. (a) The genealogical tree was created from 12 *PtrIBIs*. (b) Motif position of *PtrIBIs*. Conservative motifs are represented by differently colored boxes. The scale below the image enables estimation of the length of each nucleotide or protein sequence. (c) Conserved protein domain analysis of *PtrIBIs*. PLN02903: aminoacyl-tRNA ligase; PLN02502: lysyl-tRNA synthetase; PLN02850: aspartate-tRNA ligase; PLN02221 super family: asparaginyl-tRNA synthetase; PLN02221: asparaginyl-tRNA synthetase; PLN02603: asparaginyl-tRNA synthetase. (d) Gene structure analysis of the *PtrIBI* genes family of *P. trichocarpa*. Upstream and downstream noncoding regions are indicated by green rectangles, exons are indicated by yellow rectangles, and introns are indicated by gray lines.


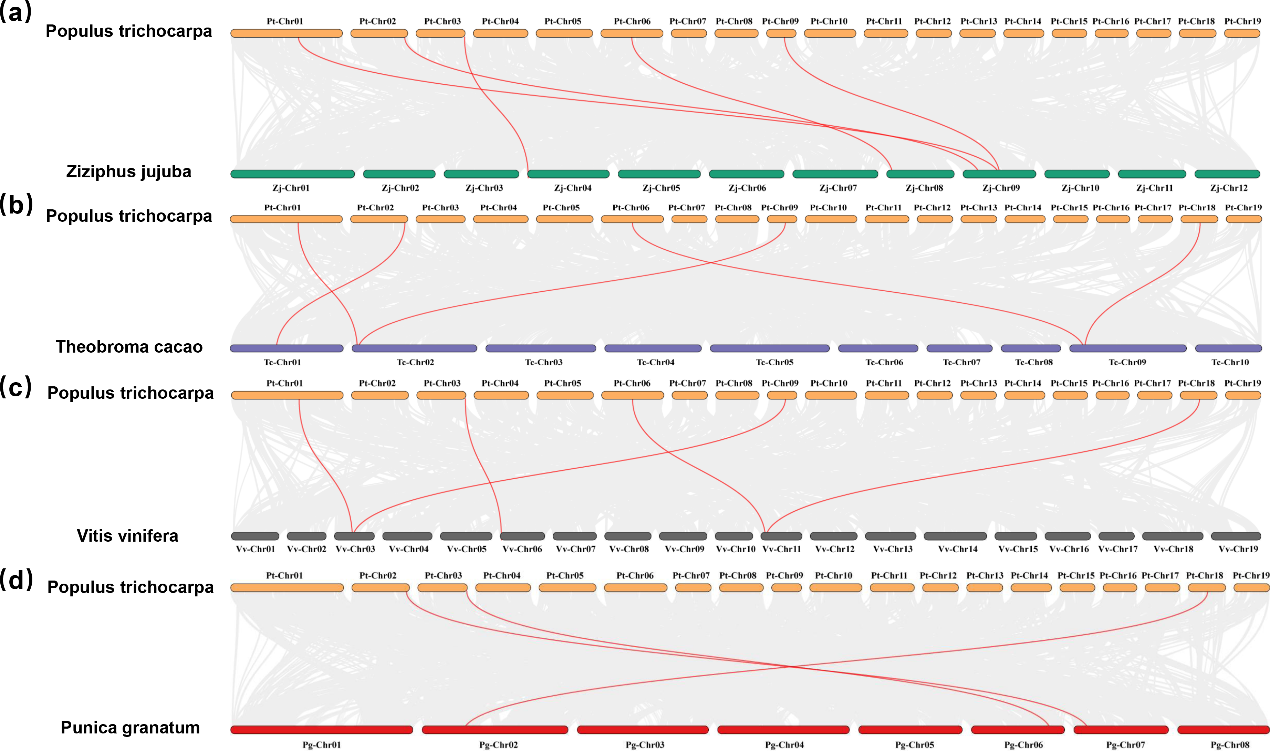

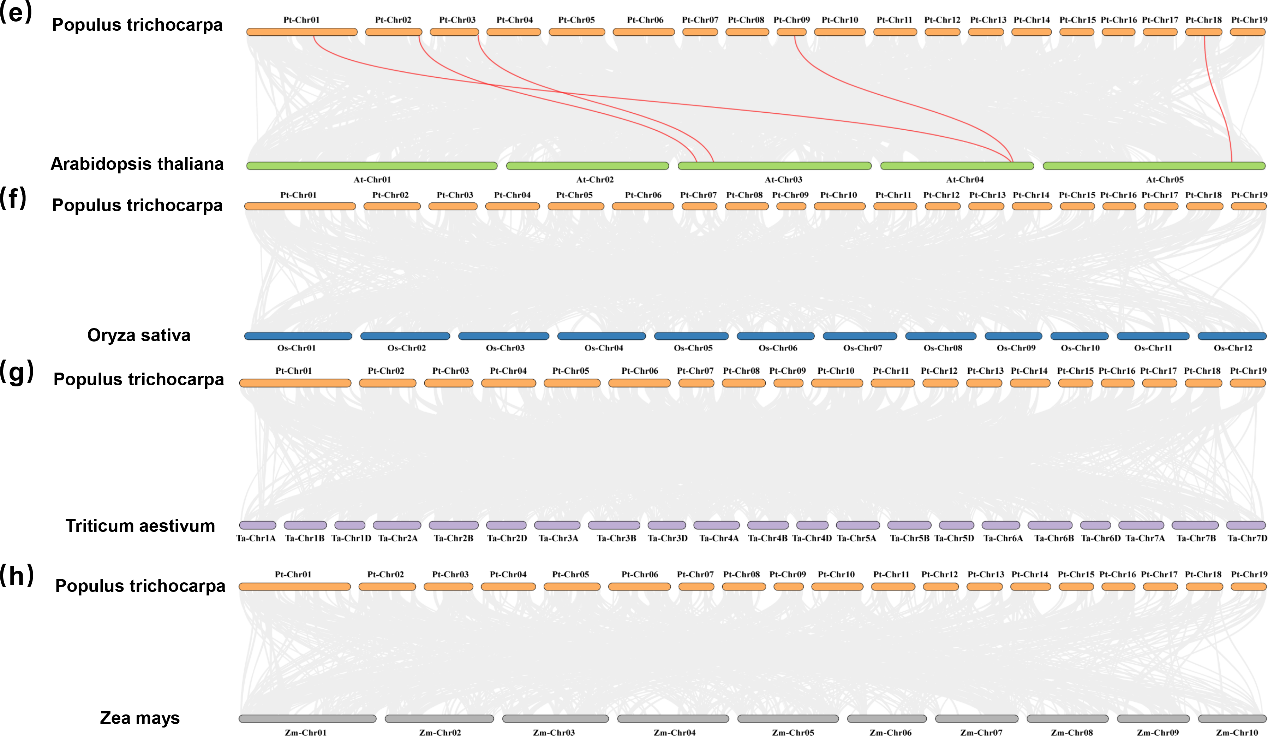


**Fig. S5.** Collinear analysis of PtrIBI genes in *P. trichocarpa* with 8 other plants. Gray lines in the background represent collinear blocks of *P. trichocarpa* and other species genomes, while red lines emphasize collinear PtrIBI genes pairs. (a) *Ziziphus jujuba*. (b) Theobroma cacao. (c) *Vitis vinifera*. (d) *Punica granatum*. (e) *Arabidopsis thaliana*. (f) *Oryza sativa*. (g) *Triticum aestivum*. (h) *Zea mays*.


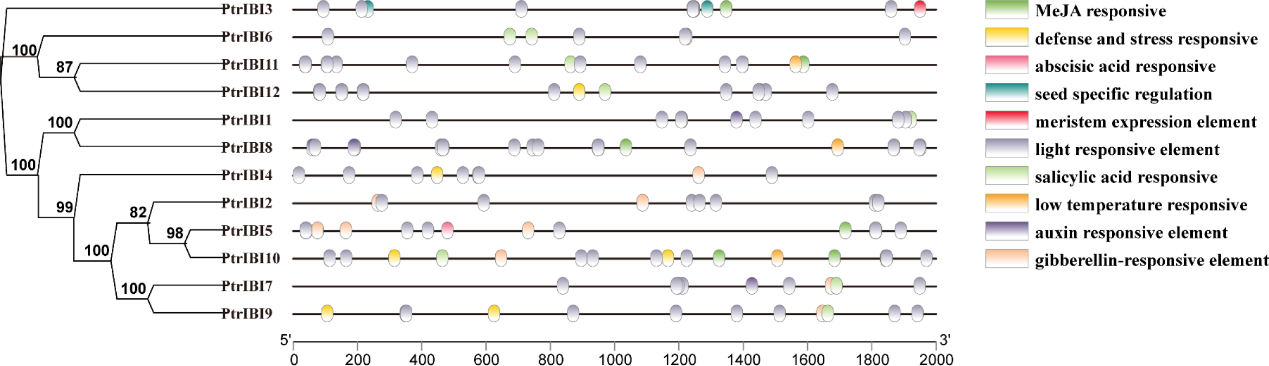


**Fig. S6.** Cis-elemental analysis in the PtrIBIs gene family of *P. trichocarpa*. Relative positions of stress and growth associated cis-elements in the promoter regions of PtrIBIs. Different colors represent different cis-acting elements, and their positions correspond to the corresponding positions of the promoters.


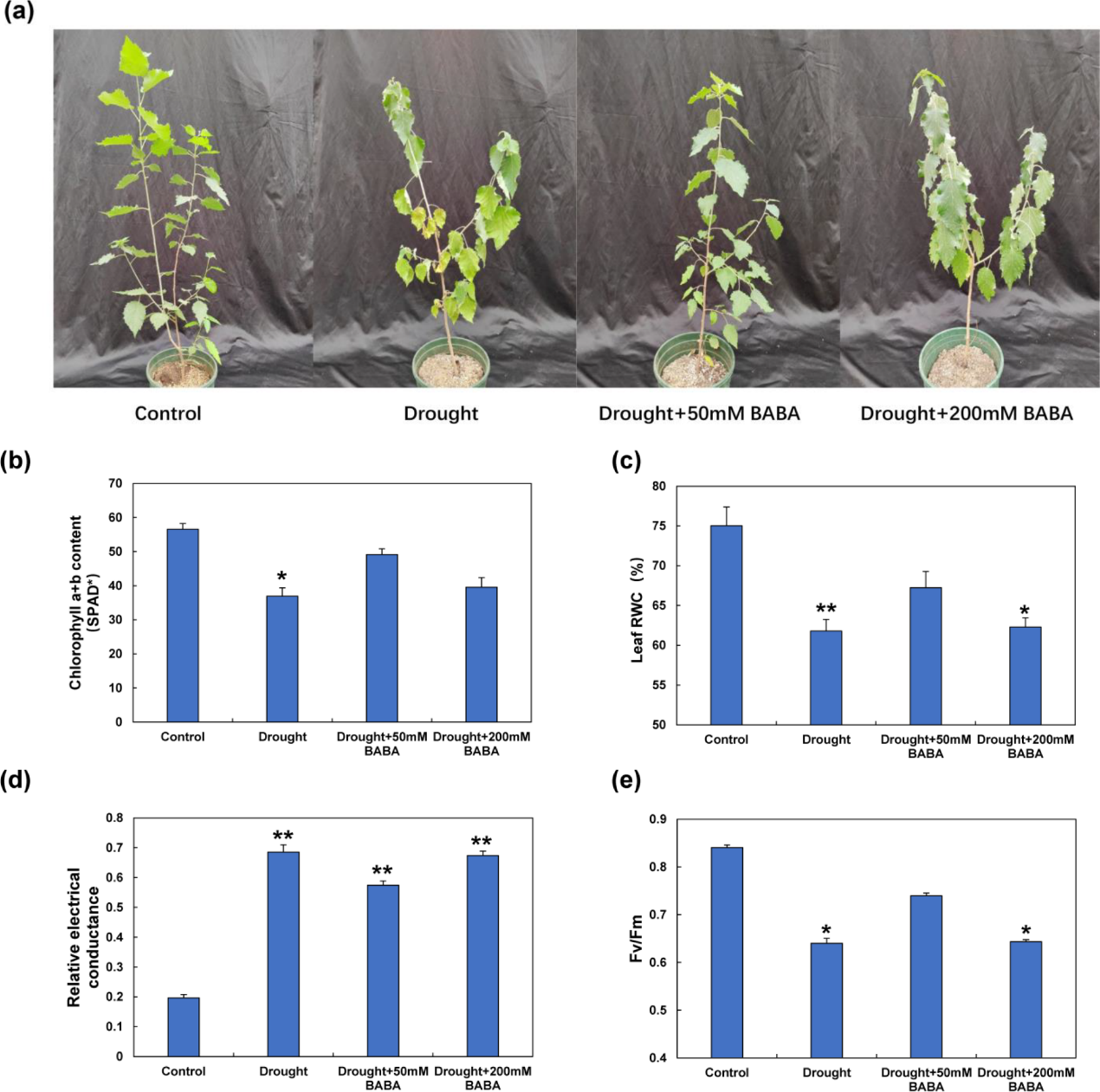


**Fig. S7.** (a) Topical application of BABA induces improved drought tolerance in *P. trichocarpa*. (b) Chlorophyll a+b content. (c) Leaf RWC. (d) Relative electrical conductance. (e) Fv/Fm.


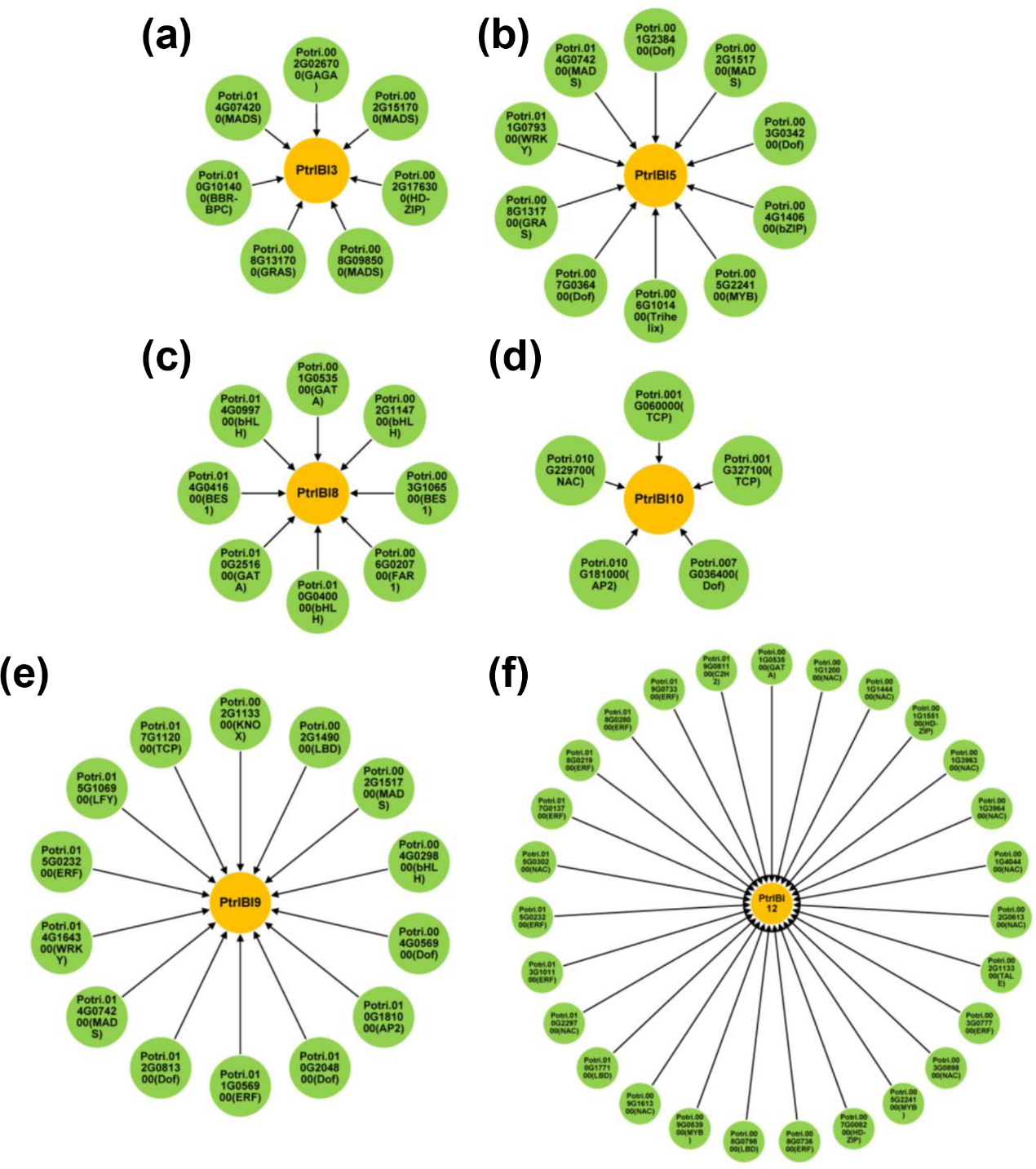


**Fig. S8.** Bioinformatic analysis of transcription factors (TFs) of PtrIBIs. (a-f) TFs-*PtrIBI3*/*5*/*8*/*9*/*10*/*12* interaction network analysis. Green circles indicate TFs upstream of PtrIBIs, and yellow circles represent *PtrIBI3*/*5*/*8*/*9*/*10*/*12.*
